# Hospitalization risk factors for children’s lower respiratory tract infection: A population-based, cross-sectional study in Mongolia

**DOI:** 10.1038/srep24615

**Published:** 2016-04-19

**Authors:** Amarjargal Dagvadorj, Erika Ota, Sadequa Shahrook, Purevdorj Baljinnyam Olkhanud, Kenji Takehara, Naoko Hikita, Bayasgalantai Bavuusuren, Rintaro Mori, Takeo Nakayama

**Affiliations:** 1 Department of Health Informatics, Kyoto University, Yoshida Konoe-cho, Syakyo-ku, Kyoto, 606-8501, Japan; 2 Department of Health Policy, National Center for Child Health and Development, 2-10-1 Okura, Setagaya-ku, Tokyo, 157-8535 Japan; 3 Department of Environmental Health Sciences, Mongolian National University of Medical Sciences, Zorig street, Ulaanbaatar-14210, Mongolia; 4 Department of Midwifery and Women’s Health, The University of Tokyo, 7-3-1 Hongo, Bunkyo-ku, Tokyo 113-0033, Japan; 5 Department of Pediatrics, Mongolian National University of Medical Sciences, Zorig street, Ulaanbaatar-14210, Mongolia

## Abstract

This study aimed to assess the potential risk factors for lower respiratory tract infection (LRTI)-related hospital admissions in Mongolian children. A population-based cross-sectional study was conducted in rural Mongolia in 2013, and 1,013 mother–child pairs were included. Of the participating children, 38.9% were admitted to hospital with LRTIs. Home smoking, low birthweight, being a male child, exclusive breastfeeding and healthcare-seeking behaviour showed substantial association with LRTI-related hospital admissions. Number of cigarettes smoked by family members showed a dose-response relationship and increased hospital admissions. Strategies to prevent second-hand-smoke exposure from adult smokers, especially inside the home, are crucial to preventing LRTI-related hospital admissions for children in Mongolia. Improving rates of exclusive breastfeeding and increasing birthweight have great potential to decrease the likelihood of children acquiring a LRTI. Educational initiatives are also necessary for women who are less likely to seek out care for their children’s symptoms.

Pneumonia in children younger than 5 years of age is responsible for 18.4% of mortality worldwide, accounting for an estimated 1.4 million deaths in 2010^1^. Furthermore, more than 20% of total neonatal deaths in developing countries are caused by serious infections every year^2^, of which 50% are caused by neonatal pneumonia^3^.

In Mongolia, respiratory tract infections (RTIs) account for one-third of communicable diseases and are the second leading cause of death in children under 5 years of age^4^. RTI-associated hospital visits in <5-year-old Mongolian children comprised 87% of all hospital visits in 2010^5^. In developed countries, up to 18% of paediatric hospital admissions were attributable to lower respiratory tract infection (LRTIs)^6^. While hospitalization for LRTI is well documented in developed countries, such data is yet to be reported from many developing countries such as Mongolia. Additionally, children in developing countries have an average five episodes of LRTI in a single year, which makes up 50% of all paediatric visits and 30% of all admissions^7^. Repeated episodes of RTI may also have a negative impact on a child’s social and cognitive development^8^.

Known risk factors for respiratory infection are reported to be mainly second-hand smoke, low socioeconomic status, sex, preterm birth, mineral deficiency and day-care attendance^9^,^10^. In 2010, LRTI in children with second-hand smoke exposure caused an estimated >165,000 deaths globally^11^. In Mongolia, national estimates indicate that tobacco smoking is highest among male adults of 15 years or older (43%) compared to females (5.2%)^12^ and approximately one in two people (42.9%) is exposed to second-hand smoke at home^13^. Furthermore, due to the nomadic culture of people in remote communities and the long distances to health facilities, it is difficult for women in rural Mongolia to seek emergency healthcare services. In this context, therefore, it is likely that women seek care advice regarding their children’s health and illnesses from people within their immediate community, such as relatives, neighbours, traditional healers, and monks. As a result, it is probable that in the absence of appropriate and timely healthcare seeking, children’s illnesses such as LRTI could become severe. Currently, information such as about the associated risk factors for LRTI in the Mongolian context is severely lacking. It is therefore crucial to identify the potential risk factors contributing to LRTI in children and associated hospital admission in order to suggest appropriate prevention strategies.

In this study, we aimed to assess potential risk factors for LRTI-related hospital admissions in Mongolian children aged 3 years old.

## Methods

### Subjects

A population-based, cross-sectional study was conducted in Bulgan, a rural province of Mongolia, from July 2013 to October 2013. The study area comprised approximately 50,000 km^2^ and a widely distributed population of about 53,000 people. This province was selected for the study because of its fairly even ratio of nomadic and sedentary residents, which is highly generalizable to other parts of the country. This study included all children aged 3 years who were born in 2010, and their mothers, who were resident in Bulgan during the study period. Lists of residents were acquired from the Bulgan registration centre, and research assistants living in each administrative area who had knowledge of the movements of nomadic residents and their current location conducted data collection. Based on this procedure, 1,083 mother-and-child pairs were eligible for this study. Women who refused to participate in the study and those who were not available during the period of data collection were excluded, which resulted in 1,019 mothers and 1,013 children being included. Details of the recruitment procedure are clarified in a previously published^14^ descriptive study of this dataset.

### Data collection and variables

The questionnaire was designed to assess the risk factors contributing to the hospital admission of children with LRTI, and was initially pilot-tested among 15 women who lived in the study field. Trained research assistants then distributed the pre-tested structured questionnaire to all participants. Women who were not able to read were still able to answer all questions as the research assistants read out the questions to them. Data on socioeconomic status, number of family members, nomadic status, smoking place, number of cigarettes smoked per day, healthcare-seeking behaviour, and breastfeeding duration were collected by face-to-face questionnaire during a door-to-door survey. Records of child birthweight and hospitalization for LRTI were transcribed from the Maternal and Child Health Handbook^15^, and anthropometric measurements on weight and height of the mother and child were taken during the door-to-door survey.

The Maternal and Child Health Handbook is provided by the government to mothers during their first antenatal health check-up with a general practitioner. The general practitioner is expected to explain to the expectant mothers how to use the handbook, and is responsible for recording antenatal check-ups, delivery process, neonatal status and measurements, infant medical check-ups, vaccination records, and history of diseases until the age of 5 years. Mothers are responsible for checking normality of growth and developmental milestones for their children and are free to take advice written in the handbook.

We asked mothers the question, “Whom do you seek for care first when your child becomes ill?” We categorized answers as formal healthcare-seeking behaviour if mothers answered “hospital physician” and “pharmacist”. For informal healthcare-seeking behaviour, we included “traditional healer”, “monk”, “partner”, and “relatives”. Both formal and informal healthcare-seeking behaviour translated to the presence of healthcare-seeking behaviour. If mothers answered “no one” to the question, we took this as an absence of healthcare-seeking behaviour.

A hospital admission for LRTI was defined as being equivalent to having severe clinical pneumonia, bronchiolitis, bronchitis, exacerbation of asthma, and viral-induced wheeze necessitating hospital admission. As Mongolian clinicians adhere to guidelines for the Integrated Management of Childhood Illness^16^, the general criteria for LRTI-related hospital admission is having cough or difficulty breathing and having any of the following signs — any danger signs, chest indrawing or stridor in a calm child. Diagnosis of LRTIs was made by local professional physicians and recorded in the maternal and child health handbook. Those who did not have this handbook were identified through the questionnaire answered by the mothers. Hospital admission was defined as admission to any hospital in or outside the province since birth till the time of survey. All admitted episodes for the condition were registered during this 3-year period.

The study setting included 20 primary-level health centres and a secondary hospital, the Bulgan Central Hospital. Details of the healthcare system are described elsewhere^14^. Basic health services for children up to 16 years old are fully subsidized by the government, and the average immunization coverage for each province in the country ranges from 94.0% to 98.9%^17^.

### Statistical analysis

Statistical analysis was performed using Stata version 13.0 (StataCorp LP, College Station, Texas, USA) on study participants of 1,019 mothers and 1,013 children.

The basic characteristics of study participants were described using the chi-squared test. Multivariable analyses were performed for the potential risk factors contributing to children being admitted to hospital for LRTI, including maternal age and education, wealth index, overall smoking (any family member), place of smoking, number of cigarettes smoked per day by any family member, nomadic status, family crowding, healthcare-seeking behaviour, sex of the child, exclusive breastfeeding, low birthweight, and use of stoves. Multicollinearity between variables was assessed using Pearson’s correlation coefficients. Calculation of the wealth index is explained elsewhere^14^ based on previous validated study methods^18^,^19^.

The main model was adjusted for all the characteristics described above: maternal age and education, wealth index, place of smoking, nomadic status, family crowding, healthcare-seeking behaviour, sex of the child, exclusive breastfeeding (≧4 months), low birthweight (≦2,499 g), and use of stoves. Since the number of cigarettes smoked is suggested to be a better predictor of smoking intensity^20^, the model was further adjusted for number of cigarettes smoked per day by any family member. The final model was interpreted using adjusted odds ratios (AORs) and 95% confidence intervals (CIs) at a statistically significant level of *p*-value < 0.05.

The population attributable fraction (PAF) was estimated by using the formula PAF = p (θ−1)/ θ, where p = the proportion of cases who were exposed, θ = AOR^21^. The PAF determines the proportional reduction in LRTI-related hospitalization of children, which hypothetically would occur if children were not exposed to a risk factor.

### Ethical Considerations

This study protocol was approved by the Ethics Committee of the National Center for Child Health and Development, Japan, as well as the Institutional Scientific Board of the National Center for Maternal and Child Health and the Ethics Committee of the Ministry of Health, Mongolia. The study protocol complied with the principles of the Declaration of Helsinki^22^ and written informed consent was obtained from women prior to study recruitment. This written informed consent included approval from mothers on behalf of their children. All data were analysed anonymously.

## Results

Data was collected from 1,019 mothers and their 1,013 children, with a high response rate of 93.8%. Tables 1 and 2 show the distribution of characteristics for all participants.

In our sample, most women were between 25 to 39 years old and were educated to secondary school level. In the sample, 45.2% of participants were from nomadic households, and 49.2% had at least one smoking family member living with them. Of the smoking households, only 3 mothers were smokers and 67.8% of smoking family members smoked inside the home. The majority of participants (95.4%) used stoves in their homes.

Of the 1,013 children in the study, 4.2% were born with a low birthweight, and most (93.6%) were exclusively breastfed in their first 4 months of life. Overall, 395 children (38.9%) had an LRTI-related hospital admission. Discrepancies reported in these numbers from an earlier descriptive study^14^ were due to missing information on LRTI-related hospital admissions.

In the multivariable model, home smoking (AOR: 1.51; 95% CI: 1.11**–**2.07), low birthweight (AOR: 2.15; 95% CI: 1.06**–**4.35), and being a male child (AOR: 1.34; 95% CI: 1.00**–**1.76) showed a positive effect. Meanwhile, exclusive breastfeeding at ≧4 months (AOR: 0.43; 95% CI: 0.24**–**0.74) and an absence of healthcare-seeking behaviour (AOR: 0.33; 95% CI: 0.17**–**0.61) were found to be negative predictors of children’s hospital admission. Use of stoves did not show any association with the outcome in the multivariable analysis (Table 3).

Number of cigarettes showed a dose-response relationship (1–10 cigarettes per day AOR: 1.34; 95% CI: 0.99–1.81 and ≧11 cigarettes per day AOR: 1.46; 95% CI: 0.98–2.18), although the strength of evidence was weak. In addition, overall smoking increased hospital admission probability by 1.38 times (95% CI: 1.04**–**1.81; not shown in table).

Concerning the confounding risk factors, the estimated PAF was 14.7% for cigarette smoking inside the home, 33.1% for low birthweight, and 40.6% for the absence of healthcare-seeking behaviour. These figures represent the reduction in LRTI-related hospitalization of children that would occur if the respective risk factors were reduced to ideal levels.

## Discussion

Our study found that children exposed to home smoking, children with a low birthweight, and children who were exclusively breastfed for less than 3 months had higher LRTI-related hospital admissions. In addition, being a male child and an absence of any formal or informal healthcare-seeking behaviour from the mother showed strong associations with LRTI-related hospital admissions. Furthermore, hospital admissions increased if family members smoked a higher number of cigarettes per day. Despite the dose-response relationship, the daily number of cigarettes smoked by family members had a weak association with LRTI-related hospital admissions (p < 0.10), probably because of the study’s small sample size. However, this may imply that controlling the number of cigarettes may motivate parents to smoke less, and reduce children’s exposure to second-hand smoke and the risk of respiratory disease.

Previous research has consistently shown the harmful effect of home smoking among all population groups, especially children^11^,^23^. Furthermore, a Swedish study found that outdoor smoking was associated with a lower urine cotinine level in children (AOR: 1.99; 95% CI: 1.1–3.6)^24^. In the current study, a parallel finding that favours outdoor smoking was also observed in the Mongolian context. However, living in a smoking household raises further concerns for Mongolian children as a great majority of Mongolia’s population lives in a *ger* — a traditional circular house without any walls inside to divide the space into separate rooms. Furthermore, it has been suggested that harmful particles are likely to be spread around the house via the smoker’s fingers^25^. Given these factors, smoking cessation among adults in the family might be the most useful strategy for protecting children from exposure, as outdoor smoking can only provide moderate protection^26^.

Our results were consistent with much of the evidence from previous studies showing that low birthweight is strongly associated with child respiratory diseases^10^. This was further supported by the PAF of 33.1% implying the importance of preventing low birthweight with regard to reducing LRTI-related hospitalization. In light of extensive evidence^27^,^28^, adoption of prenatal care integrated with useful strategies to support psychosocial factors that promote women’s reproductive health, such as low-dose aspirin, bed rest, and maternal oxygen therapy, could prevent low birthweight in Mongolia.

It is well known that exclusive breastfeeding reduces the incidence of LRTI compared to partial and no breastfeeding 29. In our study, there was a reduced risk for LRTI-related hospitalization in children who were exclusively breastfed for the first 4 months of life (p < 0.003). This result provides further evidence to support the pivotal role of exclusive breastfeeding in preventing various infectious diseases among children^30^. In addition, hand washing and improved nutrition are recommended as useful preventive measures to protect <5-year-old children from developing LRTIs^31^.

In this study, being a male child increased the likelihood of hospital admission. This finding is consistent with previous studies that have reported on the tendency of boys to have more LRTIs than girls^32^,^33^. Furthermore, in Mongolian culture, boys are perceived to be strong and so less likely to get sick^34^. This might lead to parents’ neglect of their boys, causing boys to acquire severe respiratory disease necessitating hospital admission.

Interestingly, women who refrained from seeking any care during their child’s illness had less likely to admit their children to hospital for LRTI (p < 0.001) compared to women who sought informal or formal care. In a study conducted in Pakistan, lack of maternal perception and knowledge of child illness was associated with delayed consultation^35^. This indicates that in rural Mongolia, it is probable that some mothers who are reluctant to seek health care might be putting their child’s overall health at greater risk, including worsening their child’s LRTI status. Furthermore, the PAF for the absence of healthcare-seeking behaviour was estimated at 40.6%, suggesting a need to educate mothers to seek professional health care at an early stage of their children’s respiratory illness.

A key strength of this study is the collection of population-level primary data from door-to-door surveys and from the Maternal and Child Health Handbook in a remote rural province of Mongolia. Our study is unique in that it covered an area of approximately 50,000 km^2^ and we sought data from a widely distributed population of 53,000 people. To the best of our knowledge, this is the first study in Mongolia to focus on hospital admission rates and potential risk factors associated with children’s LRTI.

Our study has several limitations. First, the data covered only the first 3 years of a child’s life after birth. Nevertheless, we succeeded in determining all LRTI episodes since their birth and that the risk of LRTI is usually highest in the early years of life. Second, other potential risk factors of LRTI-related hospital admissions might have been overlooked in our investigation, such as the type of fuel used in household stoves, nutrition, antenatal cigarette smoke exposure, and nursery attendance, where children might contract an infection from others^26^,^31^.Therefore, caution should be taken in interpreting these study findings.

In addition to home smoking, domestic solid fuel stoves are a major source of indoor air pollution in Mongolia^36^. In recent years, air pollution levels of the capital city Ulaanbaatar have exceeded WHO recommendations by 23 times^37^, and much of this pollution is due to high seasonal fuel use during extremely low temperatures, e.g. −40 °C^38^. In addition to our findings, high rates of seasonal air pollution should also be considered in relation to LRTI, as most households in this study — and in Mongolia generally^37^ — use a stove.

Given the environmental challenge of air pollution, LRTI should be recognized as a critical child health problem that may escalate in future and place a heavy burden on the national health system. In addition, suitable preventive measures are necessary to reduce pollution and improve respiratory health. In our study, more than one-third of children were admitted for LRTI, which also suggests that programs addressing early management of LRTI, especially in rural areas, would provide significant benefits to reduce the financial burden on the health system and other resource usage, as LRTI is associated with increased healthcare utilization and related cost of care^39^.

It is also crucial to increase maternal education and awareness of environmental factors associated with LRTI, and the importance of prevention and control of this condition. A population-based study that involved 288 Inuit children in Greenland reported that the population-attributable risk of LRTI associated with second-hand smoke was 47.1%^40^. Although our result (14.7%) is comparatively smaller, it could still be used to initiate education on the deleterious effects of family members’ smoking.

Since smoking and exclusive breastfeeding are modifiable lifestyle practices, increasing the awareness of second-hand smoke risks and promoting second-hand smoke prevention practices — such as reduced smoking, smoking outside and smoking cessation — as well as promoting exclusive breastfeeding practices, can improve children’s respiratory health in Mongolia. Additionally, initiatives to prevent low birthweight are necessary to decrease the likelihood of LRTIs in young children.

In conclusion, our study highlights the finding that LRTI-related hospital admissions among children might be reduced by their family members’ efforts to stop smoking, especially inside the home. Future studies should investigate specific methods of treatment and potential prevention strategies of modifiable risk factors for LRTI-related hospital admissions.

## Additional Information

**How to cite this article**: Dagvadorj, A. *et al*. Hospitalization risk factors for children’s lower respiratory tract infection: A population-based, cross-sectional study in Mongolia. *Sci. Rep.* **6**, 24615; doi: 10.1038/srep24615 (2016).

## Figures and Tables

**Table 1:** Basic characteristics of women.

**Women**	**Category**	**Distribution (%)**	**No admission for LRTIs**		**Admission for LRTIs**		***p*****value**
			**n**	**(%)**	**n**	**(%)**	
Age	18–24	169 (17.2)	99	58.6	70	41.4	0.717
	25–39	742 (75.5)	456	61.5	286	38.5	
	≧40	72 (7.3)	42	58.3	30	41.7	
	Missing (%)		36 (3.5)				
Educational level	No education	13 (1.3)	7	53.9	6	46.2	0.852
	Primary	78 (7.9)	49	62.8	29	37.2	
	Secondary	661 (66.8)	407	61.6	254	38.4	
	Tertiary	238 (24.0)	141	59.2	97	40.8	
	Missing (%)		29 (2.8)				
Wealth index	Poorest	199 (20.4)	127	63.8	72	36.2	0.644
	Poor	194 (19.7)	120	61.9	74	38.1	
	Middle	196 (19.9)	123	62.8	73	37.2	
	Wealthy	197 (20.0)	112	56.9	85	43.2	
	Wealthiest	197 (20.0)	118	59.9	79	40.1	
	Missing (%)		36 (3.5)				
Family crowding	≦4	597 (60.2)	359	60.1	238	39.9	0.498
	≧5	395 (39.8)	246	62.3	149	37.7	
	Missing (%)		27 (2.6)				
Nomadic status	Nomadic	448 (45.2)	286	63.8	162	36.2	0.247
	Sedentary	487 (49.2)	285	58.5	202	41.5	
	Seasonal	55 (5.6)	33	60.0	22	40	
	Missing (%)		29 (2.8)				
Use of stove	Yes	947 (95.4)	585	61.8	362	38.2	0.029
	No	46 (4.6)	21	45.7	25	54.4	
	Missing (%)		26 (2.5)				
Smoking place	No smoking	501(50.8)	324	64.7	177	35.3	0.051
	Inside the home	330 (33.4)	186	56.4	144	43.6	
	Outside	156 (15.8)	93	59.6	63	40.4	
	Missing (%)		26 (2.5)				
Number of cigarettes smoked per day	0	506 (50.9)	327	64.6	179	35.4	0.055
	1~10	340 (34.2)	197	57.9	143	42.1	
	≧11	147 (14.9)	82	55.8	65	44.2	
	Missing (%)		26 (2.5)				
Healthcare-seeking behaviour	Formal	742 (79.4)	432	58.2	310	41.8	0.002
	Informal	122 (13.1)	76	62.3	46	37.7	
	None	70 (7.5)	56	80.0	14	20.0	
	Missing (%)		85 (8.3)				

**Table 2:** Basic characteristics of children.

**Children**	**Category**	**Distribution (%)**	**No admission for LRTIs**		**Admission for LRTIs**		***p*****value**
			**n**	**(%)**	**n**	**(%)**	
Sex	Male	529 (52.2)	309	58.41	220	41.59	0.077
	Female	484 (47.8)	309	63.84	175	36.16	
	Missing (%)	0					
Low birthweight	≧2500 g	971 (95.8)	602	62	369	38	0.002
	≦2499 g	42 (4.2)	16	38.1	26	61.9	
	Missing (%)	0					
Exclusive breastfeeding	≦3 months	65 (6.4)	29	44.62	36	55.38	0.005
	≧4 months	946 (93.6)	588	62.16	358	37.84	
	Missing (%)		2 (0.2)				

**Table 3:** Multivariable predictors of LRTI admission.

**Predictors**	**Category**	**n**	**Adjusted odds ratio**	**95% CI**	***p*** **value**
Maternal age category	25–39	742	1		
	18–24	169	1.05	0.72–1.53	0.808
	≧40	72	1.03	0.59–1.76	0.901
Maternal education	Tertiary	238	1		
	No education	13	1.05	0.25–4.34	0.947
	Primary	78	1.10	0.58–2.05	0.777
	Secondary	661	1.07	0.72–1.56	0.740
Wealth index	Wealthiest	197	1		
	Poorest	199	1.16	0.50–2.63	0.731
	Poor	194	1.31	0.58–2.93	0.519
	Middle	196	0.93	0.53–1.60	0.793
	Wealthier	197	1.08	0.68–1.71	0.739
Smoking place	No smoking	501	1		
	Inside the home	330	1.51	1.11–2.07	0.009
	Outside	156	1.11	0.75–1.65	0.615
Nomadic	Seasonal	55	1		
	Yes	448	0.75	0.35–1.61	0.463
	No	487	1.09	0.56–2.11	0.796
Family crowding	≦4	597	1		
	≧5	395	0.88	0.65–1.18	0.387
Healthcare-seeking behaviour	Formal	742	1		
	Informal	122	0.80	0.52–1.20	0.282
	None	70	0.33	0.17–0.61	<0.001
Sex of the child	Female	484	1		
	Male	529	1.34	1.01–1.76	0.039
Exclusive breastfeeding	≦3 months	65	1		
	≧4 months	946	0.43	0.24–0.74	0.003
Low birthweight	≧2500 g	971	1		
	≦2499 g	42	2.15	1.06–4.35	0.033
Use of stove	no	46	1		
	yes	953	0.58	0.28–1.15	0.120

(n = 915) Model adjusted for variables in this table.
